# Dose-dependent erythema nodosum after initiation of semaglutide

**DOI:** 10.1016/j.jdcr.2024.02.014

**Published:** 2024-02-29

**Authors:** Jeffrey Sobieraj, Brian Schapiro, Wasim Nasir

**Affiliations:** a Department of Internal Medicine, Hurley Medical Center, Flint, Michigan; b Division of Dermatology, CTA Pathology, Ann Arbor, Michigan; c Division of Dermatology, Dermatology and Cosmetic Center, Flint, Michigan

**Keywords:** erythema nodosum, Ozempic, semaglutide

## Introduction

Erythema nodosum (EN) is an acute septal panniculitis characterized by tender erythematous nodules primarily localized on extensor surfaces of the lower legs. EN is considered a hypersensitivity response to antigenic stimuli.^1^ The exact mechanism is disputed, with some authors postulating the pathogenesis as due to deposition of immune complexes in the venules of the septae of the subcutaneous fat^1^^,^^2^ Though the etiology is typically idiopathic, it can occur with a vast number of underlying causes including streptococcal infections, tuberculosis, sarcoidosis, Behçet disease, inflammatory bowel disease, medications, and pregnancy.^1^ Medications that have been most commonly associated with EN include sulfonamides, bromides, and oral contraceptives, as well as a host of vaccines. Semaglutide (Ozempic) is a glucagon-like peptide-1 analog used in the treatment of type 2 diabetes, and more recently, it has gained considerable popularity as a treatment for weight loss.^3^ Here we report a unique case of EN attributed to semaglutide.

## Case report

**Fig 1:**
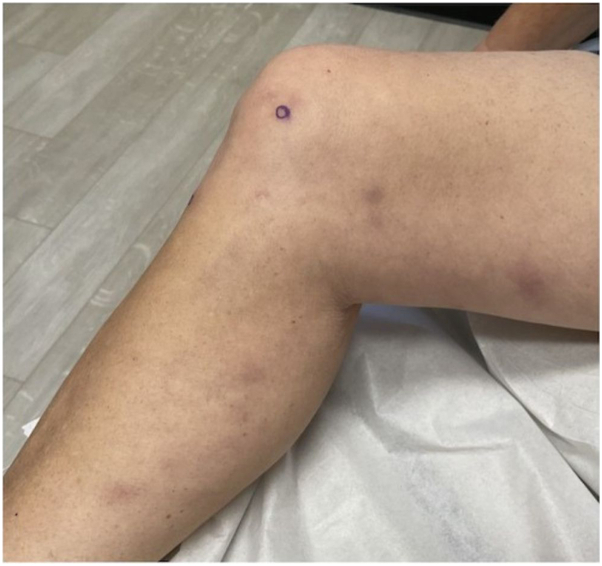
Tender, erythematous, and warm subcutaneous nodules involving the right medial calf, knee, and thigh.

**Fig 2:**
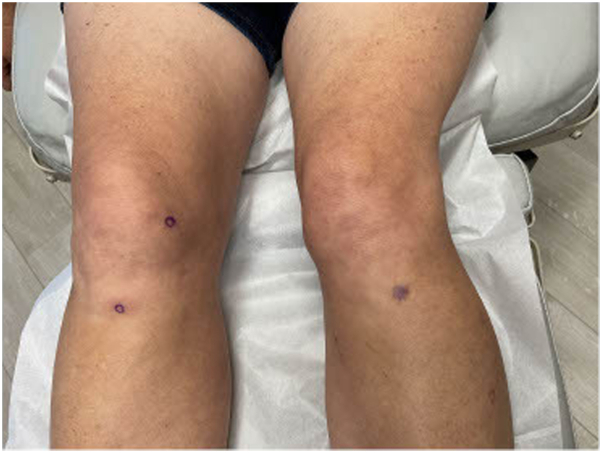
Tender, erythematous, and warm subcutaneous nodules involving the bilateral anterior knee.

**Fig 3:**
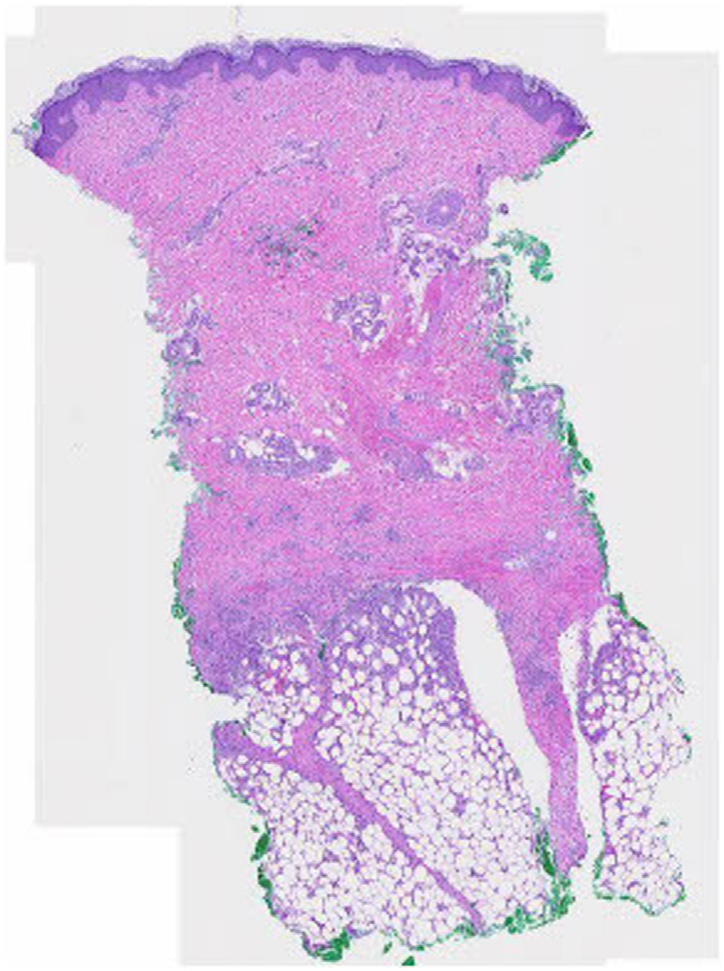
Sections show a normal epidermis and dermis with underlying pannicular septal fibrosis with an inflammatory cell infiltrate which extends into the periphery of the pannicular lobules.

**Fig 4:**
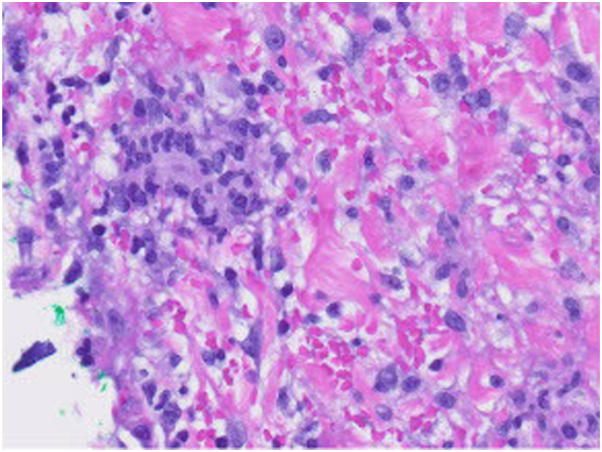
Sections show a lymphohistiocytic infiltrate in the pannicular septum with giant cell formation.

A 56-year-old woman on semaglutide for weight loss presented to an outpatient dermatology clinic for a 4-month history of ill-defined erythematous and tender warm nodules and plaques distributed over her bilateral lower extremities. Her medical history was significant for type 2 diabetes, hypertension, hypothyroidism, gout, hyperlipidemia, arthritis, asthma, atopic dermatitis, and seasonal allergies, and her medications at the time of presentation were levothyroxine, allopurinol, atorvastatin, claritin, semaglutide, fish oil, daily multivitamin, vitamin D, and vitamin E. Her only new medication was the initiation of semaglutide 9 months prior to onset of symptoms. Her initial dose was 0.25 mg weekly. Her primary care physician increased her dose to 1 mg weekly and she developed her first lesion 3 weeks after this dose increase. Her semaglutide dose was increased again to 2 mg 1 month later. Following this dose increase, she developed a significant increase in the number, size, and tenderness of the lesions. Her semaglutide dose was decreased back to 1 mg 3 months after symptoms onset due to repeated episodes of hypoglycemia. Since this dose-decrease, she reported a significant reduction in skin lesions. Prior to presentation at our clinic, she was in her usual state of health. Her review of systems was negative, including no recent fever, fatigue, night sweats, lymphadenopathy, chest pain, shortness of breath, abdominal pain, hematochezia or melena, leg swelling, or any oral or genital ulcers. Exam revealed multiple tender, erythematous, and warm subcutaneous nodules involving her bilateral lower extremities (Figs 1 and 2). Punch biopsies of 2 separate lesions, one on her right anterior knee and the other on her right anterior lower leg, demonstrated widened subcutaneous septa with fibrosis associated with a lymphohistiocytic infiltrate with giant cells, some of them with clear clefts (asteroid bodies). The inflammatory cell infiltrate extended into the periphery of the pannicular lobules (Figs 3 and 4). These results were consistent with EN. Complete blood count and comprehensive metabolic panel were within normal limits. Antistreptolysin O titer was negative. Chest x-ray was negative.

## Discussion

EN classically presents as tender, warm, erythematous nodules on the anterior lower extremities. An estimated 30% to 50% of cases are idiopathic, but there are also a wide range of etiologies. It occurs more often in women between 25 and 40 years^2^ EN tends to resolve in 2 to 6 weeks without any scar or atrophy. The patient had no medical history to suggest a known etiology for EN.

We believe this case highlights a novel presentation of EN secondary to semaglutide. Evidence supports a hypersensitivity response to antigenic stimuli as the pathogenesis for EN. Semaglutide, a peptide molecule, may serve as the antigenic stimulus in the formation of immune complex depositions leading to EN lesions. Length of onset of EN following initiation of new medication can widely vary from days to months.^4^^,^^5^ Semaglutide is the only new medication that was started prior to onset of symptoms. Furthermore, there was a strong dose-dependent effect of semaglutide on the patient’s lesions that suggests a correlation between initiation of semaglutide and development of EN. At the most recent visit, the patient remained on a 1 mg weekly dose and was still developing sporadic lesions despite being outside the window of the expected resolution of symptoms attributed to idiopathic causes. This dose is the same dose in which she first noticed the EN lesions, which suggests an ongoing trigger for development of EN lesions. As her semaglutide dose was further titrated down, the intensity, number, and duration of lesions significantly decreased. On her current low dose of semaglutide, she continues to have intermittent scattered lesions, though they are tolerable. We recommended possibly discussing alternative weight loss medications with her primary care physician if her symptoms become more bothersome. As semaglutide gains popularity for its effect on weight loss, providers must consider this potential trigger when investigating and managing EN cases.

With some authors postulating the pathogenesis as due to deposition of immune complexes in the venules of the septae of the subcutaneous fat.

## Conflicts of interest

None disclosed.
